# Genotyping and diversity analysis of local avocado landraces in La Palma, Canary Islands

**DOI:** 10.3389/fpls.2025.1572870

**Published:** 2025-04-16

**Authors:** Verónica Pérez, Nerea Larranaga, M. Librada Alcaraz, J. Ignacio Hormaza

**Affiliations:** ^1^ Jardín de Aclimatación de La Orotava (JAO), Instituto Canario de Investigaciones Agrarias (ICIA), Puerto de la Cruz, Spain; ^2^ Laboratorio de Agrobiología Juan José Bravo Rodríguez (Cabildo de La Palma), Unidad Técnica del Instituto de Productos Naturales y Agrobiología (IPNA-CSIC), Santa Cruz de La Palma, Spain; ^3^ Instituto de Hortofruticultura Subtropical y Mediterranea La Mayora (IHSM La Mayora - CSIC - UMA), Algarrobo-Costa, Spain

**Keywords:** genetic diversity, genotyping, germplasm, genetic erosion, microsatellites, molecular markers, simple sequence repeats (SSRs)

## Abstract

Although both informal and formal avocado breeding has been carried out for over a century, current commercial avocado production worldwide is concentrated on only a few cultivars, with ‘Hass’ dominating the global avocado market. This narrow genetic base hinders the long-term sustainability of the crop amid the challenges derived from climate change. For this reason, studying the existing avocado diversity is essential and, in this work, we address this issue by analyzing the genetic diversity of 177 avocado trees from La Palma, Canary Islands, and comparing it with 89 avocado accessions maintained at a worldwide avocado germplasm bank in Málaga, Spain. In the Canary Islands, particularly on the island of La Palma, local avocado germplasm can be found due to the historic commercial and cultural ties with America that have lasted for over 500 years. Currently, isolated avocado trees that originated from or descended from these transoceanic movements still persist. Using nine SRR molecular loci, we characterized these isolated trees that have adapted to insular conditions and often predate commercial varieties. The analyses grouped the samples by racial origin and revealed a high percentage of interracial hybrids, likely resulting from seeds exchange between farmers and free pollination.

## 1 Introduction

The avocado (*Persea americana* Mill.) is a subtropical evergreen woody perennial fruit tree crop within the genus *Persea* of the Lauraceae family. It is among the most economically important subtropical/tropical fruits globally. Its commercial volume has increased significantly in recent years worldwide driven mainly by its health benefits and rising consumer demand (Dreher and Davenport, 2013; Schaffer et al., 2013). Currently, avocados are cultivated on over 900,000 ha across more than 65 countries, with a production increase of 30% in the five last years, producing more than 8 million tons annually (FAOSTAT, 2024). Despite extensive cultivation and resulting higher production levels, the global avocado market is dominated by a single variety, ‘Hass’, which originated from a chance seedling nearly a century ago in California (Crane et al., 2013; Dreher and Davenport, 2013; Chen et al., 2009) and patented in 1935. The commercial success of ‘Hass’ began in the 1970s (Bost et al., 2013), attributed to its favorable transport characteristics that facilitated its global expansion, and as well as its appealing organoleptic qualities. Avocados are native to Mesoamerica, where they have been consumed for over 10,000 years (Galindo-Tovar et al., 2008; Chen et al., 2009). *P. americana* is a polymorphic species comprising at least eight botanical varieties or subspecies that evolved under diverse edaphoclimatic conditions and geographical isolation (Chen et al., 2009). Among these, three, referred to as horticultural races, are of agronomic importance: Mexican (*P. americana* var. *drymifolia*), Guatemalan (*P. americana* var. *guatemalensis*) and West Indian (*P. americana* var. *americana*). The West Indian race originated in lowland tropical regions, the Guatemalan race in the valleys of Central American mountain ranges, and the Mexican race in the highland tropics of Southern Mexico (Bergh, 1992). These races exhibit distinct ecological adaptations: the Mexican and Guatemalan races are better adapted to cooler subtropical climates, with the Mexican race being more cold-tolerant than the Guatemalan race, while the West Indian race is more adapted to warmer tropical climates. Recently, a new Colombian group closely related to the West Indian race has been identified in South America (Berdugo-Cely et al., 2023). No reproductive barriers exist between the avocado races (Alcaraz and Hormaza, 2009; 2014), and intercrossing between them has resulted in the wide diversity of avocados that exists today (Chanderbali et al., 2008). Most commercially grown avocado varieties in subtropical and Mediterranean climates are interracial hybrids between Guatemalan x Mexican races. West Indian avocados have lower oil content in the fruit compared to avocados from the other races (Gómez-López, 2000), and different volatile chemical profiles at the ripening (Pereira et al., 2013).

The avocado was introduced to the Canary Islands, likely including La Palma, by traders from Central America during the 19th century (Pérez and Sagot, 1867; Parrilla-González, 2007), mainly of West Indian varieties. However, some authors suggest an earlier introduction (García Cabezon, 1965; García-Medina and Bello-González, 2015) soon after 1492, with seeds being brought multiple times during the early days of transatlantic exploration, as many ships traveling between Spain and the Americas stopped at the islands, although the first documented introduction occurred in 1835. During that year, avocado seeds, likely of the West Indian race, were sent from Havana, Cuba, to be planted at the Jardín de Aclimatación de La Orotava in Tenerife, the largest of the Canary Islands (García Cabezon, 1965; Machado, 2014). These avocados were likely propagated from seeds and planted as isolated trees in back gardens or along the edges of orchards with other tropical or subtropical crops, and were used primarily for domestic consumption (García Cabezon, 1965; Galán-Saúco and Fernández-Galván, 1983; 1985). Around the 1950s, fruits of quality and characteristic of the West Indian race were observed and described in Tenerife by Popenoe (1959). Nowadays, these avocados are locally known as “summer avocados” because the fruit ripens in the summer months, or as “criollo avocados” and “aguacates del País” (*avocados from the country*) in reference to their origin. It was not until 1953, when efforts began in Tenerife to introduce commercial varieties from California and Florida, such as ‘Fuerte’, ‘Hass’, ‘Rincon’, or ‘Bacon’, which are interracial hybrids primarily derived from crosses between Mexican and Guatemalan avocados. Subsequent crosses between these introduced varieties and local germplasm of West Indian origin resulted in new local varieties, such as ‘Orotava’, ‘Gema’ or ‘Robusto’ (García Cabezón, 1963; 1965). Commercial avocado production began in the 1970s in La Palma with the introduction, probably from the neighboring island of Tenerife, of the most important commercial varieties at the time, like ‘Mc Arthur’, ‘Anaheim’, ‘Rincón’, ‘Zutano’, ‘Topa topa’, ‘Nabal’ or ‘Orotava’, according to unpublished lab documents from Dr. Juan Jose Bravo Rodríguez, an agricultural researcher who promoted avocado cultivation in La Palma during these years. Some of these varieties were reported to be present in the Canaries by Arteaga Eiriz and Odriozola Azurmendi (1969). This initial production and trade focused on varieties with thin skin, which had limited shelf-life and poor transport qualities, leading to a decline in commercialization in subsequent years. Although references are limited, growers attribute the decline also to the emergence of *Phytophthora cinnamomi*, a root disease that devastated the crop. For years, avocado cultivation on La Palma was largely forgotten, with the fruit being consumed only locally. However, avocado production resurged in recent decades, driven by the global increase in consumption and higher market prices. In the Canary Islands, a ban has been in place since the 1980s on importing certain tropical fruits, including avocados, to prevent the introduction of exotic pests (BOE, 1987). Consequently, only locally produced avocados are consumed, shielding the regional market from competition with avocados imported from other regions. Currently, 2,500 ha of avocado are cultivated in the Canary Islands, mainly in La Palma (1,100 ha), Tenerife (950 ha), Gran Canaria (300 ha), and La Gomera (44 ha) with the remaining area distributed among the other islands (ISTAC, 2024). This accounts for a significant portion of the more than 20,000 hectares of avocados cultivated in Spain (MAPA, 2023). Commercial production is concentrated on ‘Hass’ and ‘Fuerte’ cultivars, with ‘Fuerte’ harvested between November and February and ‘Hass’ from October to May.

The long history of avocado cultivation in the Canary Islands, particularly on La Palma, suggests that this isolated population may harbor unique genetic diversity resulting from centuries of adaptation to local conditions. Given the island’s 500-year history of cultural and commercial exchange with the Americas, we hypothesized that La Palma’s avocado population would exhibit significant genetic differentiation from commercial varieties and contain valuable interracial hybrids that could contribute to broadening the genetic base of cultivated avocados. To test this hypothesis, in this study, we performed a molecular characterization of the diversity of La Palma local avocados focusing on those considered as old by local residents using nine SSRs loci in order to enhance our understanding of the avocado heritage in La Palma, which is crucial for its conservation. Microsatellite markers have been extensively utilized to characterize diversity in avocado (Schnell et al., 2003; Alcaraz and Hormaza, 2007; Gross-German and Viruel, 2013; Guzmán et al., 2017; Boza et al., 2018; Cañas-Gutiérrez et al., 2019; Sandoval-Castro et al., 2021; Ruiz-Chután et al., 2023; Yangaza et al., 2024). While new generation molecular markers are also available for avocado germplasm characterization (Ge et al., 2019b; Kuhn et al., 2019; Talavera et al., 2019), SSR markers remain valuable for this kind of studies since they are easy to use and have reference data available.

## 2 Materials and methods

### 2.1 Plant material

**Figure 1:**
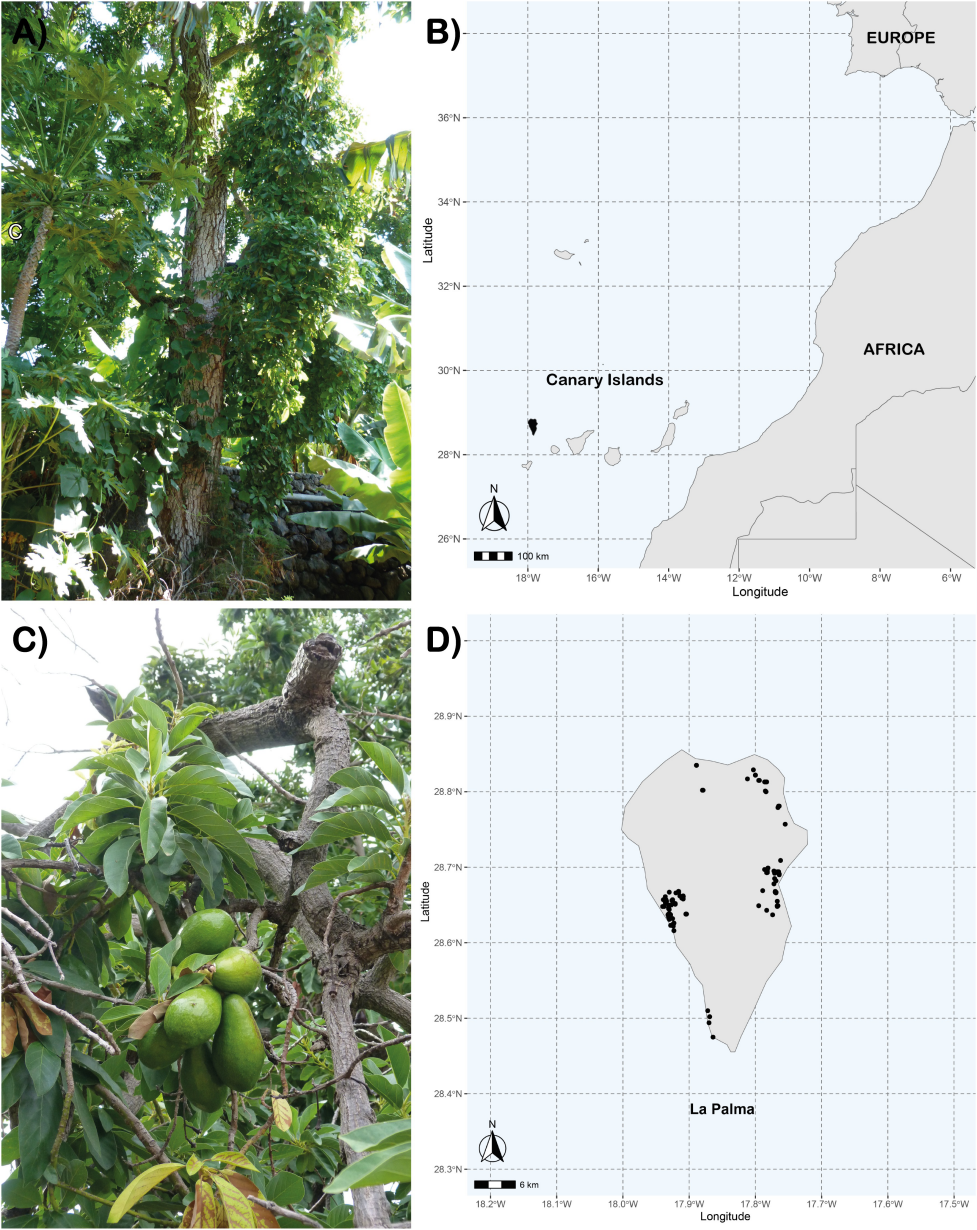
**(A, C)** Images of some sampled old avocados, **(B)** geographical location of the Canary Islands and La Palma, and **(D)** dots representing avocado trees prospected in La Palma Island.

The Canary Islands is an archipelago composed of seven volcanic islands belonging to Spain, located in the Atlantic Ocean (29° 24’ 40’’ N, 27° 38’ 16’’W) about 97 km off the coast of Morocco that is part of the Macaronesia, a biogeographic area composed of four archipelagos in the North Atlantic Ocean (Azores, Canary Islands, Cape Verde and Madeira). The easternmost islands (Lanzarote, Fuerteventura), are geologically older, have lower elevations, and are more arid, while the westernmost islands (Gran Canaria, Tenerife, La Gomera, La Palma and El Hierro), are geologically younger, higher in elevation and more humid. Significant avocado cultivation is found only on these westernmost islands, except El Hierro (ISTAC, 2024). This study analyzed 177 *Persea americana* samples collected from different localities on La Palma, Canary Islands, during 2018 (**Figure 1**). The sampling was conducted based on prior information from two previous studies (Martín-García, 1996; Pérez-Hernández, 2011). Additional information was obtained from governmental agronomic agencies, the La Palma Agrodiversity Center (CAP), and consultations with local residents to ensure comprehensive coverage of avocado diversity in the island. A reference avocado collection with 89 accessions from IHSM La Mayora-UMA-CSIC (IHSM Collection) previously characterized with SSR molecular markers by Alcaraz and Hormaza (2007) was used for comparison.

### 2.2 DNA extraction, amplification and fragment sizing

Plant DNA extraction was performed from young leaves that were preserved in plastic bags with silica gel until DNA extraction, following a modified cetyl trimethylammonium bromide (CTAB) method (Hormaza, 2002). DNA concentration was quantified using a BioDrop µLite spectrophotometer (Biochrom US, Holliston, MA, USA). Genomic DNA was amplified with nine selected SSR loci (Sharon et al., 1997; Ashworth and Clegg, 2003) (**Table 1**). PCR amplification was performed in a final volume of 15µl containing 16mM(NH_4_)_2_SO_4_, 67 mM Tris-HCl pH 8.8, 0.01% Tween-20, 20.3 mM MgCl_2_, 0.1 mM of each dNTP, 0.3 µM of each primer, 20 ng of genomic DNA, and 1 unit of Biotaq™ DNA polymerase (Bioline, London, UK). Forward primers were labelled with WellRed fluorescent dyes on the 5’ end (Sigma-Aldrich, St. Louis, MO, USA). Reactions were performed on a thermocycler (Bio-Rad Laboratories, Hercules, CA, USA) with the following temperature profile (Alcaraz and Hormaza, 2007): an initial step of 2 min at 94°C, 35 cycles of 45 s at 94°C, 45 s at 55°C, and 1 min at 72°C, and a final step of 5 min at 72°C. PCR products were separated by capillary electrophoresis in a CEQ™ 8000 capillary DNA analysis system (Beckman Coulter, Fullerton, CA, USA). Samples were denaturalized at 90°C for 120s, injected at 2.0 Kv for 30s, and separated at 6.0 Kv for 35 min. A range of samples were used as positive controls to guarantee size accuracy and to minimize run-to-run variation.

**Table 1:** Repeat motif of the Simple Sequence Repeat (SSR) loci used in this study.

Locus name	Repeats
AVAG21*^1^*	(TC)_24_
AVD001*^2^*	(CT)_12_
AVD003*^2^*	(CT)_19_
AVD006*^2^*	(TC)_9_(AC)_19_
AVD013*^2^*	(AG)_7_, (GA)_3_, (TCT)_4_
AVD017*^2^*	(TC)_18_, (AC)_8_
AVO102*^2^*	(GA)_12_
AVMIX03*^1^*	(TG)_16_, (AG)_20_
AVMIX04*^1^*	(AG)_12_, (CAA)_5_, (ACAG)_10_

^1^ Sharon et al. (1997); ^2^ Ashworth et al. (2003).

### 2.3 Fingerprinting, genetic diversity and population structure analyses

La Palma avocado samples were classified according to their collection area: north, south, east and west. The following genetic indices were calculated for both the La Palma samples and the reference collection: number of alleles, number of private alleles, observed heterozygosity (Ho), expected heterozygosity (He) and Wright´s fixation indices (Fis) using the adegenet (Jombart, 2008) and the popgenreport (Adamack and Gruber, 2014) packages implemented in R software. The results obtained were used to cluster the La Palma samples among themselves and in relation to the reference collection. For this, a similarity matrix using the Bruvo coefficient was generated to create a UPGMA (Unweighted Pair Group Method with Arithmetic Mean) clustering. Additionally, the genetic structure of both groups was analyzed using STRUCTURE software (Hubisz et al., 2009), with a “burn-in” of 20.000 steps, and 200.000 Monte Carlo Markov Chain replications and 50 interactions for values of *k* ranging from 1 to 10. To consolidate the results, all interactions were combined using the *large k greedy* algorithm using CLUMPAK software (Kopelman et al., 2015).

### 2.4 Genetic erosion

Five years after the initial data collection, the sampled avocado trees were revisited to assess their survival status and, when possible, determine the cause of death for those that were no longer alive. For trees that could not be physically visited, their presence was verified through Google Earth (2024) as their large size facilitated identification.

## 3 Results and discussion

Despite extensive research on avocado germplasm worldwide (Lahav et al., 2024), there remains a significant gap in our understanding of the genetic diversity of isolated avocado populations in regions outside its center of origin in the Americas. An example is the Canary Islands, where centuries of adaptation may have produced unique genetic variants. Our work addresses this knowledge gap by providing the first comprehensive genetic characterization of La Palma’s avocado population.

### 3.1 Diversity indices

A total of 177 samples of old avocado trees, morphologically identified as belonging to the West Indian race or as old trees, collected on La Palma, Canary Islands, were genotyped using nine SSR loci and compared with a reference collection of 89 avocado accessions. The La Palma samples were classified based on the geographical origin in west, south, east and north. Most samples were collected from the western and eastern regions of the island. The sample size was smaller in the southern region, where banana cultivation predominates, and avocado trees are primarily found as isolated individuals along the borders of banana orchards. Nonetheless, samples from this region were included to ensure representation of avocados from across the entire island. Although more samples were collected from the northern region than from the south, the overall number was still lower compared to the eastern or western regions of the island. The northern region of the island is characterized by a colder climate, which is less suitable for these avocado genotypes that are better adapted to warmer conditions. However, this area was included in the study because it hosts large avocado trees, whose seeds have traditionally been used as seedling rootstocks for commercial cultivars, as reported by local residents. In La Palma and other Canary Islands, it is common practice for growers to use West Indian genotypes as rootstocks for the production of commercial cultivars (Hernández Delgado and Méndez Hernández, 2020). The 177 samples from La Palma exhibited a total of 161 alleles, including 69 private alleles. In comparison, the IHSM avocado collection, consisting of 89 accessions, contained 150 alleles of which 61 were private. The larger size samples from La Palma likely explains the higher number of total and private alleles. However, these data also highlight the significant genetic diversity among La Palma´s old local avocados. This diversity may be attributed to the greater representation of West Indian (WI) genotypes in La Palma, whereas the IHSM collection predominantly comprises Guatemalan x Mexican hybrids with limited representation of WI genotypes. In both groups, the observed diversity was high with observed heterozygosity (Ho) values slightly higher in the IHSM collection (0.82) compared to the La Palma samples (0.72), while expected heterozygosity (He) was similar in both groups (0.85 for the IHSM collection and 0.80 for La Palma). For La Palma, this diversity could be attributed to the introduction of highly diverse founding genotypes. These indices are reflected in the Fis index, which was marginally lower for the IHSM collection (0.03) compared to La Palma (0.1). These findings are consistent with those of Alcaraz and Hormaza (2007), who also reported positive but low Fis values in the IHSM collection. The overall low Fis values for both La Palma and the IHSM collection indicate minimal inbreeding (**Table 2A**).

**Table 2:** Diversity indices: Number of trees (N° trees), number of alleles (N° alleles), number of private alleles (N° private alleles), observed heterozygosity (Ho), expected heterozygosity (He) and fixation index (Fis).

A) La Palma and IHSM La Mayora Collection.						
Samples	N° trees	N° alleles	N° private alleles	Ho	He	Fis
La Palma	177	161	69	0.723	0.802	0.098
IHSM collection	89	150	61	0.821	0.851	0.034
B) La Palma: west, south, east and north.						
Area	N° trees	N° alleles	N° private alleles	Ho	He	Fis
West	78	111	22	0.717	0.772	0.07
South	4	40	1	0.861	0.829	-0.042
East	73	116	24	0.69	0.782	0.114
North	22	89	15	0.828	0.818	-0.01

Avocado samples were grouped based on their collection locations as previously described, and genetic indices were calculated for each group (**Table 2B**). The number of samples in each group was uneven due to selection criteria prioritizing trees that were either old, descended from old trees, or exhibited characteristics of the Western Indian race. As a result, some geographical areas had greater representation than others. Specifically, the western (78 trees) and eastern (73 trees) regions together accounted for 85.3% of all samples. Correspondingly, the total number of alleles was higher in these regions, with 116 alleles identified in the east and 111 alleles in the west, where sampling was more extensive. Similarly, the number of private alleles was higher in these regions, with 24 private alleles identified in the east and 22 in the west. Observed and expected heterozygosity were similar across all groups. However, the fixation index (Fis) showed slight variations. The eastern and western regions had positive values (0.11 and 0.07, respectively). In contrast, the northern and southern regions had negative values (-0.01 and -0.04, respectively) likely reflecting the smaller sample size in these regions. Comparing these results with other studies using SSR markers in avocado is challenging due to differences in the loci analyzed and in the number of samples. For example, Schnell et al. (2003) analyzed 428 avocado plants from the National Germplasm Repository (NGR) in Miami (394 plants) and a set of 34 clones from the University of California South Coast Field Station (SCFS) using 14 SSR and identified 256 alleles, and reported an average observed heterozygosity (Ho) of 0.64. Similarly, Alcaraz and Hormaza (2007) analyzed 75 accessions, some of which were included in this study, with 16 SSR markers and identified 156 different amplification fragments, also reporting a Ho of 0.64. In Tanzania, Juma et al. (2020b) examined 226 local avocado trees grown from seeds in the Southern highlands using 10 SSR loci, identifying 167 alleles and reporting Ho and He values of 0.84 and 0.65, respectively. In a separate study, Yangaza et al. (2024) examined 270 accessions from the northern region of the country using 11 SSR loci, identifying 197 alleles and reporting Ho and He values of 0.58 and 0.72, respectively.

### 3.2 Genetic clustering between La Palma and a world avocado collection

**Figure 2:**
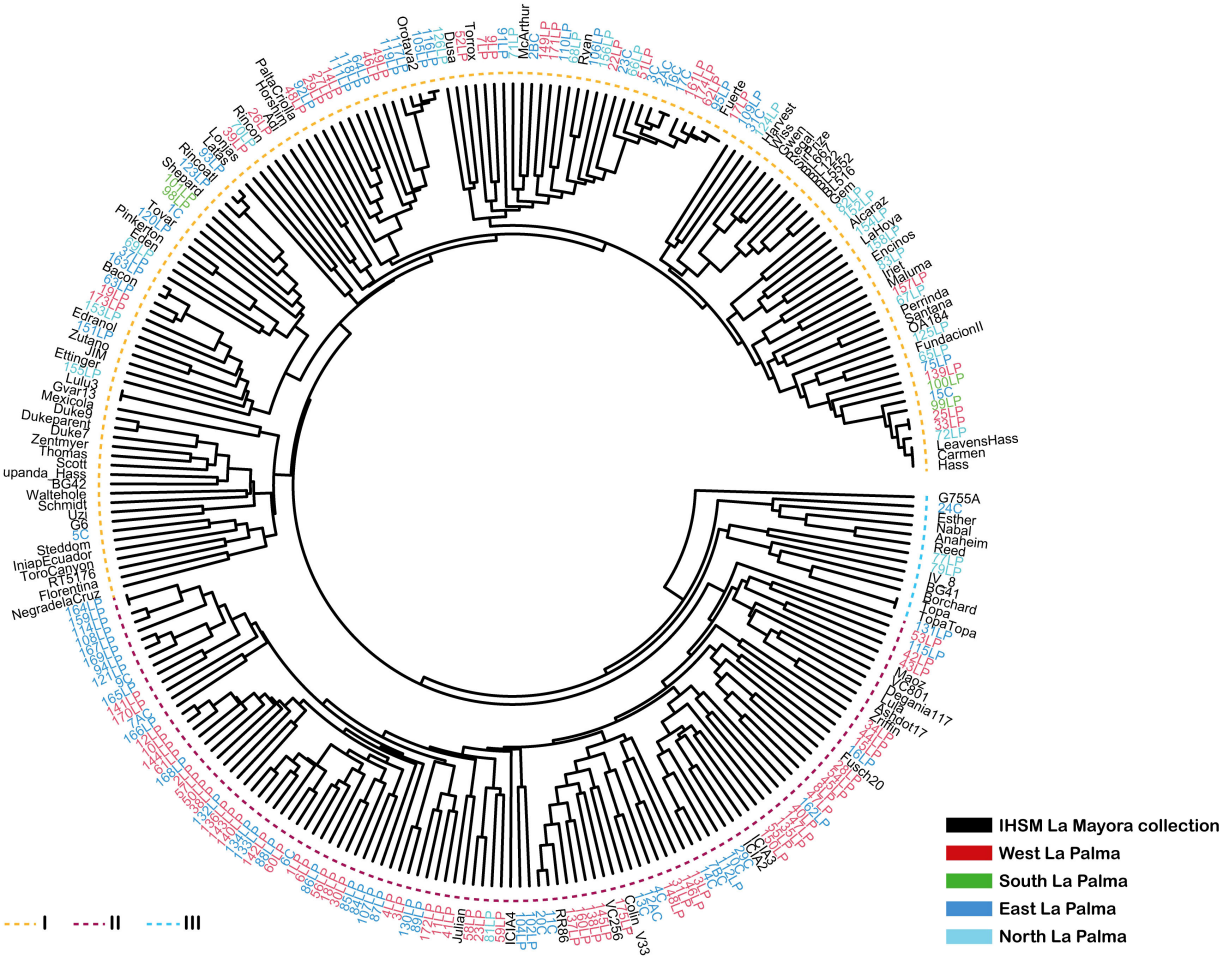
UPGMA dendrogram based on the Bruvo similarity index, illustrating the relationship between La Palma samples and IHSM La Mayora avocado collection. The samples are represented with different colors (right legend) depending on their geographical origin in La Palma island and from the *ex situ* collection maintained at the IHSM La Mayora in mainland Spain. The two main branches generated, related with racial origin, are indicated with a dotted line with different colors (left legend).

Samples from La Palma were compared with the IHSM avocado collection and grouped using UPGMA clustering with a Bruvo distance approximation. The results, shown in **Figure 2**, revealed two primary branches or main clusters and a group of additional samples at the base of the tree. One of the branches (I) predominantly consists of genotypes from the IHSM collection, with some La Palma´s genotypes interspersed. Notably, genotypes from all geographical regions of La Palma are represented within this branch. For example, the four genotypes from the southern region, as well as most from the northern region, are included, suggesting that these genotypes may have a Mexican or Guatemalan origin, or represent hybrids of these races, since most of the IHSM collection accessions in this group are known to belong to these two races or their hybrids. These genotypes are likely derived from traditional commercial varieties introduced during the last century. This branch also includes genotypes from the western and eastern regions of La Palma, indicating the presence of diverse genotypes or racial groups in these areas. Within this larger cluster, a smaller branch is closely associated with ‘Fuerte’, one of the earliest cultivars introduced for commercial production in the Canary Islands (García Cabezon, 1965). Although these samples were identified as old by locals, they were introduced after the mid-20th century to assess their potential production. Another large cluster (II), located at the bottom of the dendrogram, includes only samples from the western and eastern parts of the island. These samples are associated with specific accessions from the IHSM La Mayora collection, known to have components of West Indian genotypes, such as ‘Fuchs 20’, ‘Maoz’, ‘Degania117’, ‘Ashdot17’, or ‘RR86’, as confirmed by previous studies (Alcaraz and Hormaza, 2007; Talavera et al., 2019; Cohen et al., 2023). This group also includes other genotypes associated to the West Indian race, such as ‘Julian’, ‘ICIA2’, ‘ICIA3’ and ‘ICIA4’, which were developed at the Instituto Canario de Investigaciones Agriarias (ICIA) in the Canary Islands (González-Carracedo et al., 2022). Only one sample from the northern part of the island was identified in this group. A small group of samples from La Palma in the base of tree that comprises genotypes as ‘Topa-Topa’, ‘Reed’ or ‘Nabal’ genotypes considered as Mexican or Guatemalan race or hybrids between them (Talavera et al., 2019; González-Carracedo et al., 2022). This group shows greater genetic distance from the other clusters. According to unpublished laboratory records by Dr. Juan José Bravo Rodríguez, these genotypes were introduced to La Palma, during the last century.

### 3.3 Genetic structure of La Palma and a world avocado collection

**Figure 3:**
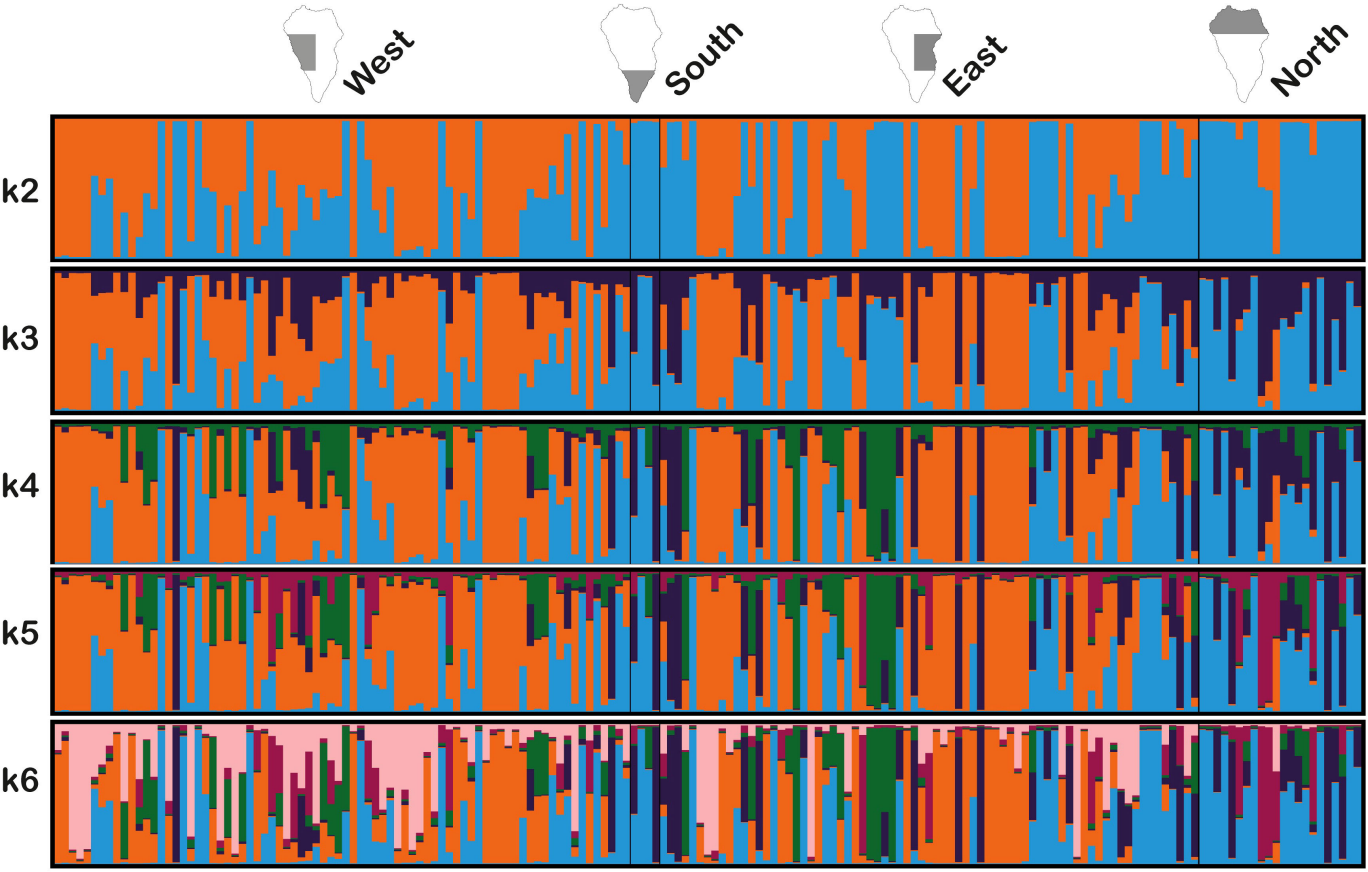
Inference of population structure based IHSM La Mayora avocado collection, 89 accessions, and 177 La Palma avocado genotypes across four geographical areas; west, south, east, and north, using Bayesian simulations for *k*=2 to *k*=6.

The IHSM La Mayora collection and the samples prospected from La Palma were analyzed using a Bayesian algorithm implemented in STRUCTURE software. This analysis revealed clear population differences between the two groups for all values of *k* tested (**Figure 3**). As the value of *k* increased, the population structure of La Palma samples remained largely consistent, primarily in the western and eastern regions of the island. However, new colors emerged in La Mayora collection and in the southern and northern regions of La Palma, indicating greater genetic differentiation among these groups of samples.

Analyzing the La Palma samples by geographical region revealed greater genetic similarity between the western and eastern regions, as well as between the southern and northern regions. This observation is consistent with the UPGMA clustering analysis, which indicates an affinity with the West Indian race in the western and eastern regions. Conversely, genotypes with Guatemalan x Mexican ancestry were more frequent in the southern and northern regions, although some were also found in the western and eastern regions. Additionally, when considering *k=*2, hybrids between both groups are observed. Based on these results, it is likely that the current diversity in La Palma avocado trees has been generated through interracial crossings facilitated by free cross-pollination over time. This finding is significant because it highlights the potential of La Palma’s diverse avocado genotypes for future agronomic evaluation. The presence of interracial hybrids has previously been documented in Canary Islands (Galán-Saúco and Fernández-Galván, 1983), especially in Tenerife (Parrilla - González, 2007). Intercrossing between genotypes has also occurred in other regions, such as Guatemala, where the historical events, ecological conditions, and reproductive traits have shaped the final diversity (Ruiz-Chután et al., 2023). Similar processes have been observed in non-native avocado growing regions, such as China (Ge et al., 2019a; Liu et al., 2020) and Tanzania (Juma et al., 2020a).

In the case of *k=2*, 18 samples from La Palma had a higher assignment probability (>0.99) to the population represented by orange color in the Bayesian analysis, which is practically exclusive for this group. The alleles of these samples were compared to those in the IHSM La Mayora avocado collection to identify possible matches with known genotypes. Although no exact allele matches were found, certain alleles at specific loci were present in samples from the collection known to be associated to the West Indian race. These alleles include: 178 and 218 in AVAG21, 228 in AVD001, 192 in AVD003, 312 and 326 in AVD006, 220 in AVD013, 177 in AVD017, 192 in AVD102, 143 and 152 in AVMIX03, and 175 in AVMIX04. This data suggests that the samples associated with the West Indian race in La Palma represent a heterogeneous group, with diverse genotypes introduced in the past. This is further supported by the morphological diversity observed in these samples.

**Figure 4:**
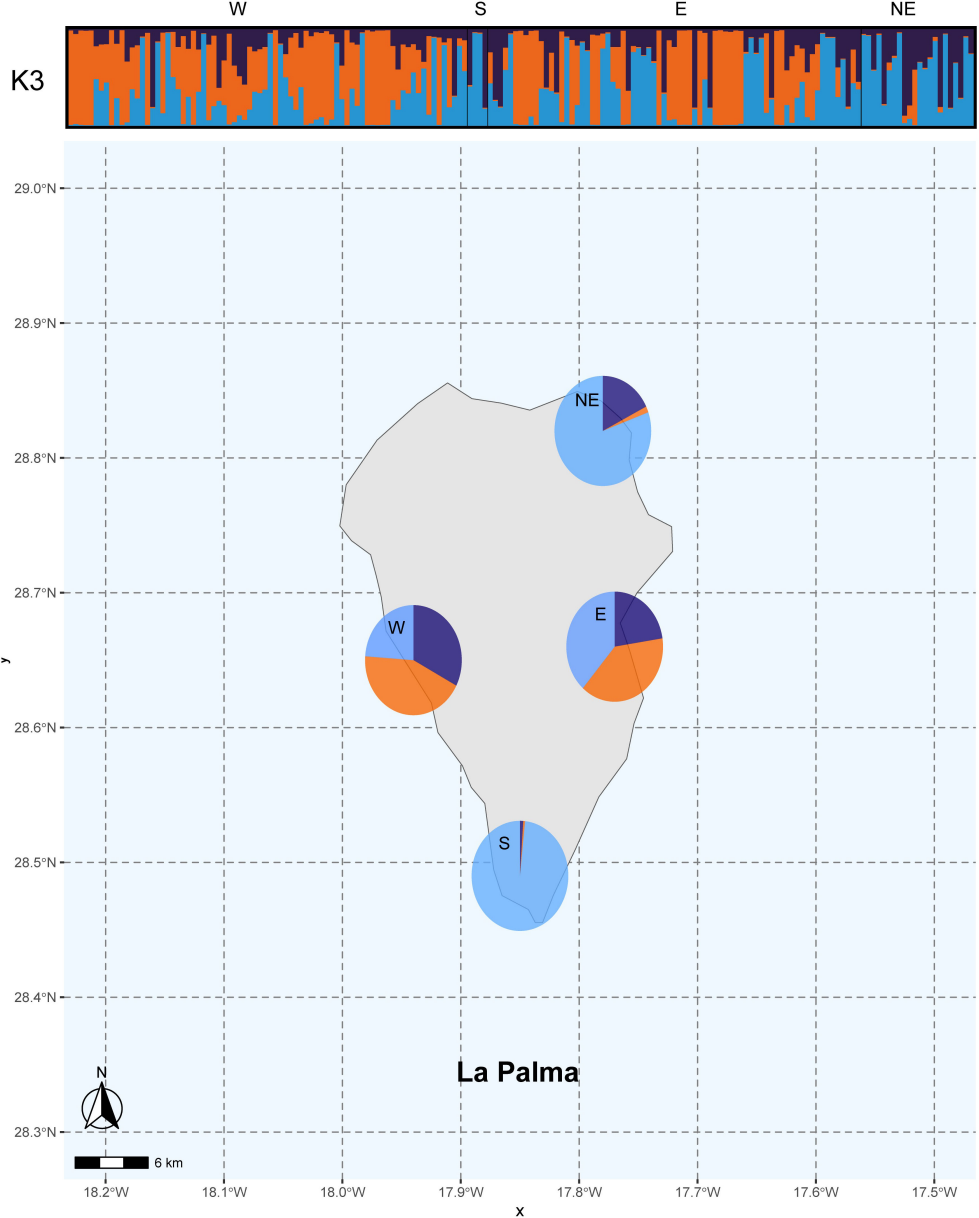
Population structure inference based on 161 alleles across 177 La Palma avocado genotypes using Bayesian simulations. CLUMPAK results (*k*= 3) for each genotypes sampled in a bar and like pies charts illustrating admixture proportions across four geographical: west, south, east and north.

The population structure for *k=3* is shown on a map of La Palma (**Figure 4**). Based on these results, the orange genetic cluster, associated with Western Indian genotypes, was detected only in the western and eastern regions of the island. These areas, with warmer climates, are suitable for these genotypes. These genotypes were frequently found in old farms, originally used for sugar cane cultivation, which were later replaced by banana crops that remain dominant today (Rodríguez-Hernández et al., 2022). Historical records suggest that farm owners with trade connections to the Caribbean or Antilles brought seeds from these regions and planted tropical trees near ports and within colonial homes (Parrilla-González et al., 2012). This included not only avocados but also species like rose apple (*Syzygium jambos* (L.) Alston), coffee (*Coffea arabica* L.), guava (*Psidium guajava* L.), mango (*Mangifera indica* L.), and other edible fruit trees (Pérez-Hernández, 2011). In contrast, the West Indian genetic component was almost absent among the analyzed genotypes in the northern and southern regions of the island, although some tall and vigorous trees, characteristic of this race, were present. This exuberance is likely due to the more humid environmental conditions in these areas. Identifying West Indian avocados or their hybrids is significant because these genotypes are traditionally used as rootstocks and are known for their tolerance to *Phytophthora cinnamomi* (Schnell et al., 2003), saline soils (Lazare et al., 2021), and irrigation with recycled water (Cohen et al., 2023).

### 3.4 Genetic erosion and conservation of old local avocado trees on La Palma island

To assess the conservation of avocado genetic resources, the trees included in the study were revisited five years later to record their survival status and to identify possible causes for any losses. Of the original 177 trees, 9 trees were no longer present, representing a 5% loss. Among these, five of these trees had been cut down, and four were buried as a result of a recent volcanic eruption (Taddeucci et al., 2023). Although volcanic events are rare, they can occasionally lead to the loss of genetic resources in affected areas. The gradual loss of avocado trees over time in La Palma is not a new phenomenon, as earlier studies have documented trees that no longer exist (Martín-García, 1996). However, despite these losses, the extant germplasm could still contribute to improve the current avocado commercial diversity. Previous studies have focused on this direction, mainly addressing tolerance to root diseases (Gallo-Llobet and Siverio, 1997). Future studies should prioritize leveraging this genetic diversity to tackle emerging challenges in avocado cultivation. These include declining irrigation water quality and availability, as well as soil degradation, which threaten the sustainability of avocado production. Harnessing the genetic potential of these traditional trees could ensure the resilience and productivity of avocado crops in changing environmental conditions.

## 4 Conclusions

This work highlights the significant avocado diversity present on La Palma island. The results indicate that this diversity is the product of the historical introduction of the three main horticultural avocado races over time followed by subsequent intercrossing among them. The West Indian race, prevalent among many old trees, exhibits specific agronomic characteristics, such as ripening timing, tolerance to saline soils, or resistance to root diseases. The hybrids generated and cultivated in La Palma, likely possess a combination of agronomic and local adaptation traits of significant interest. Therefore, evaluating this germplasm is essential to determine their full potential and suitability for addressing current agricultural challenges, such as climate resilience and sustainable farming practices. Moreover, conserving this germplasm should be a priority to ensure its availability for future avocado agricultural innovation and improvement efforts.

## Data Availability

The datasets presented in this study can be found in the digital.csic online repository by using the following link: http://hdl.handle.net/10261/384405.

## References

[B1] Adamack A. T. Gruber B. (2014). PopGenReport: simplifying basic population genetic analyses in R. Methods Ecol. Evol. 5, 384–387. doi: 10.1111/2041-210X.12158

[B2] Alcaraz M. L. Hormaza J. I. (2007). Molecular characterization and genetic diversity in an avocado collection of cultivars and local Spanish genotypes using SSRs. Hereditas 144, 244–253. doi: 10.1111/j.2007.0018-0661.02019x 18215247

[B3] Alcaraz M. L. Hormaza J. I. (2009). Selection of potential pollinizers for ‘Hass’ avocado based on flowering time and male–female overlapping. Sci. Hortic. 121, 267–271. doi: 10.1016/j.scienta.2009.02.001

[B4] Alcaraz M. L. Hormaza J. I. (2014). Optimization of controlled pollination in avocado (*Persea americana* Mill., Lauraceae). Sci. Hortic. 180, 79–85. doi: 10.1016/j.scienta.2014.10.022

[B5] Arteaga Eiriz F. Odriozola Azurmendi J. M. (1969). Variedades comerciales de aguacates, 2-69-H. Ministerio Agricult. 23 pp. Available online at: https://www.mapa.gob.es/ministerio/pags/biblioteca/hojas/hd_1969_02.pdf.

[B6] Ashworth V. E. T. M. Clegg M. T. (2003). Microsatellite markers in avocado (*Persea americana* Mill.): genealogical relationships among cultivated avocado genotypes. J. Hered. 94, 407–415. doi: 10.1093/jhered/esg076 14557394

[B7] Berdugo-Cely J. A. Cortés A. J. López-Hernández F. Delgadillo-Durán P. Cerón-Souza I. Reyes-Herrera P. H.. (2023). Pleistocene-dated genomic divergence of avocado trees supports cryptic diversity in the Colombian germplasm. Tree Genet. Genomes 19, 42. doi: 10.1007/s11295-023-01616-8

[B8] Bergh B. O. (1992). The origin, nature, and genetic improvement of the avocado. Califor. Avocado Soc Yearb. 76, 61–75.

[B9] BOE (1987). Orden de 12 de marzo de 1987 que se establecen para las islas Canarias las normas fitosanitarias relativas a la importación, exportación y tránsito de vegetales y productos vegetales. BOE n°. 72, 25 March 1987.

[B10] Bost J. Smith N. Crane J. (2013). “History, distribution and uses,” in The avocado: botany, production and uses, ed. B. Schaffer, B.N. Wolstenholme. Ed. Whiley A. W. (CABI, Wallingford, UK), 10–30.

[B11] Boza J. E. Tondo C. L. Ledesma N. Campbell R. J. Bost J. Schnell R. J.. (2018). Genetic differentiation, races and interracial admixture in avocado (*Persea americana* Mill.), and Persea spp. evaluated using SSR markers. Genet. Resour. Crop Ev. 65, 1195–1215. doi: 10.1007/s10722-018-0608-7

[B12] Cañas-Gutiérrez G. P. Alcaraz L. Hormaza J. I. Arango-Isaza R. E. Saldamando-Benjumea C. I. (2019). Diversity of Avocado (*Persea americana* Mill.) cultivars from Antioquia (Northeast Colombia) and comparison with a worldwide germplasm collection. Turk. J. Agric. For. 43, 437–449. doi: 10.3906/tar-1807-25

[B13] Chanderbali A. S. Albert V. A. Ashworth V. E. Clegg M. T. Litz R. E. Soltis D.. (2008). *Persea americana* (avocado): bringing ancient flowers to fruit in the genomics era. BioEssays 30, 386–396. doi: 10.1002/bies.20721 18348249

[B14] Chen H. Morrel P. L. Ashworth V. E. de la Cruz M. Clegg M. T. (2009). Tracing the geographic origins of mayor avocado cultivars. J. Hered. 100, 56–65. doi: 10.1093/jhered/esn068 18779226

[B15] Cohen H. Bar-Noy Y. Irihimovitch V. Rubinovich L. (2023). Effects of seedling and clonal West Indian rootstocks irrigated with recycled water on ‘Hass’ avocado yield, fruit weight and alternate bearing. N. Z. J. Crop Hortic. Sci. 51, 39–51. doi: 10.1080/01140671.2022.2098779

[B16] Crane J. H. Douhan G. Faber B. A. Arpaia M. L. Bender G. S. Balerdi C. F.. (2013). “Cultivars and rootstocks,” in The avocado: botany, production and uses. Eds. Schaffer B. Wolstenholme B. N. Whiley A. W. (CABI, Wallingford, UK), 200–233.

[B17] Dreher M. Davenport A. (2013). [amp]]lsquo;Hass’ avocado composition and potential health effects. Crit. Rev. Food Sci. Nutr. 53, 738–750. doi: 10.1080/10408398.2011.556759 23638933 PMC3664913

[B18] FAOSTAT (2024). Food and Agriculture Organization. Available online at: https://www.fao.org/faostat/en/home (Accessed September 6, 2024).

[B19] Galán-Saúco V. Fernández-Galván D. (1983). Evaluación de la población local de aguacates antillanos de la isla de la Gomera. Comunicaciones INIA. Ministerio de agricultura, Pesca y Alimentación Vol. 56 (Serie: Producción vegetal), 47.

[B20] Galán-Sauco V. Fernández-Galván D. (1985). Avocado culture in the canary islands. Calif. Avocado Soc 69, 73–80.

[B21] Galindo-Tovar M. E. Ogata-Aguilar N. Arzate-Fernandez A. M. (2008). Some aspects of avocado (*Persea americana* Mill.) diversity and domestication in Mesoamerica. Genet. Resour. Crop Evol. 55, 441–450. doi: 10.1007/s10722-007-9250-5

[B22] Gallo-Llobet L. Siverio F. (1997). Tolerancia resistencia a la podredumbre de raíz, causada por *P. cinnamomi* Rand. en patrones de la raza antillana. Phytoma Esp. 86, 31–36.

[B23] García Cabezón A. (1963). Variedades nuevas de aguacates obtenidas en Tenerife. Boletín INIA XXIII, 203–206.

[B24] García Cabezon A. G. (1965). Avocado culture in the canary islands. Calif. Avocado Soc Yaerb. 49, 47–48.

[B25] García-Medina S. Bello-González A. (2015). Proyecto Recuperación de variedades de aguacate antillano en el municipio de Mogán. Rev. Agropecuaria 22, 27–33.

[B26] Ge Y. Zang X. Tan L. Wang J. Liu Y. Li Y.. (2019a). Single-molecule long-read sequencing of avocado generates microsatellite markers for analyzing the genetic diversity in avocado germplasm. Agronomy 9, 512. doi: 10.3390/agronomy9090512

[B27] Ge Y. Zhang T. Wu B. Tan L. Ma F. Zou M.. (2019b). Genome-wide assessment of avocado germplasm determined from Specific Length Amplified Fragment sequencing and transcriptomes: population structure, genetic diversity, identification, and application of race-specific markers. Genes 10, 215. doi: 10.3390/genes10030215 30871275 PMC6471495

[B28] Gómez-López V. (2000). Fruit characterization of Venezuelan avocado varieties of medium oil content. Sci. Agric. 57, 791–794. doi: 10.1590/S0103-90162000000400031

[B29] González-Carracedo M. Alonso S. B. Brito Cabrera R. S. Jiménez-Arias D. Pérez-Pérez J. A. (2022). Development of retrotransposon-based molecular markers for characterization of *Persea americana* (Avocado) cultivars and horticultural races. Agronomy 12, 1510. doi: 10.3390/agronomy12071510

[B30] Google Earth (2024). Google Earth Pro 7.3.610201 (Mountain View, CA: Google LLC).

[B31] Gross-German E. Viruel M. A. (2013). Molecular characterization of avocado germplasm with a new set of SSR and EST-SSR markers: genetic diversity, population structure, and identification of race-specific markers in a group of cultivated genotypes. Tree Genet. Genomes 9, 539–555. doi: 10.1007/s11295-012-0577-5

[B32] Guzmán L. F. Machida-Hirano R. Borrayo E. Cortés-Cruz M. Espíndola-Barquera M. D. C. Heredia García E. (2017). Genetic structure and selection of a core collection for long term conservation of avocado in Mexico. Front. Plant Sci. 8. doi: 10.3389/fpls.2017.00243 PMC5323459 28286510

[B33] Hernández Delgado P. Méndez Hernández C. (2020). “El aguacate en Canarias,” in Cultivo, Poscosecha y Procesado del Aguacate. Eds. Namesny A. Conesa C. Hormaza I. Lobo G. (SPE3 sl, Valencia, Spain), 27–40.

[B34] Hormaza J. I. (2002). Molecular characterization and similarity relationships among apricot (*Prunus Armeniaca* L.) genotypes using simple sequences repeats. Theor. Appl. Genet. 104, 321–328. doi: 10.1007/s001220100684 12582704

[B35] Hubisz M. J. Falush D. Stephens M. Pritchard J. K. (2009). Inferring weak population structure with the assistance of sample group information. Mol. Ecol. Resour. 9, 1322–1332. doi: 10.1111/j.1755-0998.2009.02591.x 21564903 PMC3518025

[B36] ISTAC (2024). Instituto Canario de Estadística. Available online at: http://www.gobiernodecanarias.org/istac/ (Accessed September 6, 2024).

[B37] Jombart T. (2008). adegenet: a R package for the multivariate analysis of genetic markers. Bioinformatics 24, 1403–1405. doi: 10.1093/bioinformatics/btn129 18397895

[B38] Juma I. Geleta M. Nyomora A. Saripella G. V. Hovmalm H. P. Carlsson A. S.. (2020a). Genetic diversity of avocado from the southern highlands of Tanzania as revealed by microsatellite markers. Hereditas 157, 1–12. doi: 10.1186/s41065-020-00150-0 32928297 PMC7489003

[B39] Juma I. Nyomora A. Hovmalm H. P. Fatih M. Geleta M. Carlsson A. S.. (2020b). Characterization of Tanzanian avocado using morphological traits. Diversity 12, 64. doi: 10.3390/d12020064

[B40] Kopelman N. M. Mayzel J. Jakobsson M. Rosenberg N. A. Mayrose I. (2015). Clumpak: a program for identifying clustering modes and packaging population structure inferences across *K* . Mol. Ecol. Resour. 15, 1179–1191. doi: 10.1111/1755-0998.12387 25684545 PMC4534335

[B41] Kuhn D. N. Livingstone D. S. Richards J. H. Manosalva P. Van den Berg N. Chambers A. H. (2019). Application of genomic tools to avocado (*Persea americana*) breeding: SNP discovery for genotyping and germplasm characterization. Sci. Hortic. 246, 1–11. doi: 10.1016/j.scienta.2018.10.011

[B42] Lahav E. Lavi U. Hormaza J. I. (2024). “Genetics and breeding,” in The Avocado: Botany, Production and Uses. Eds. Carrillo D. Schaffer B. Whiley A. W. Wolstenholme B. N. (Wallingford, UK: CAB International), 45–75.

[B43] Lazare S. Cohen Y. Goldshtein E. Yermiyahu U. Ben-Gal A. Dag A. (2021). Rootstock-dependent response of Hass avocado to salt stress. Plants 10, 1672. doi: 10.3390/plants10081672 34451717 PMC8399844

[B44] Liu Y. Ge Y. Zhan R. Lin X. Zang X. Li Y.. (2020). Molecular markers and a quality trait evaluation for assessing the genetic diversity of avocado landraces from China. Agric 10, 102. doi: 10.3390/agriculture10040102

[B45] MaChado J. L. (2014). El Real Jardín Botánico de Aclimatación de la Orotava en sus fuentes documentales: Desde el 17 de agosto de 1788 al 17 de diciembre de 1853 Vol. 379 (Independent Publishing Platform Jose Luis MaChado).

[B46] MAPA (2023). Superficies y producciones anuales de cultivos. Available online at: https://www.mapa.gob.es/es/ (Accessed September 6, 2024).

[B47] Martín-García C. (1996). Población de aguacates antillanos en la isla de San Miguel de la Palma. [Dissertation thesis]. Tenerife: University of La Laguna.

[B48] Parrilla-González M. L. (2007). Prospección, recolección y caracterización de los aguacates antillanos de la isla de Tenerife. [Dissertation thesis]. Tenerife, University of La Laguna.

[B49] Parrilla-González M. Ríos-Mesa D. Méndez-Hernández C. Hernández-González. J. Z. Fernández-Galván D. Hernández-Delgado P. M.. (2012), 81–86. Preliminary Study of the Seedling Avocado (*Persea americana* Mill.) Population of Tenerife. Abstract retrieved from International Symposium on Banana: XXVIII International Horticultural Congress on Science and Horticulture for People 928, Lisbon, Portugal.

[B50] Pereira M. E. C. Tieman D. M. Sargent S. A. Klee H. J. Huber D. J. (2013). Volatile profiles of ripening West Indian and Guatemalan-West Indian avocado cultivars as affected by aqueous 1-methylcyclopropene. Postharvest Biol. Technol. 80, 37–46. doi: 10.1016/j.postharvbio.2013.01.011

[B51] Pérez V. Sagot P. A. (1867). de la végétation aux îles Canaries: des plantes des pays tempérés et des plantes des régions intertropicales et physionomie générale de leur agricultura. J. l´agriculture Des. Pays Chauds 1865-1866 1, 59. Paris: Challamel aîné libraire-commissionnaire.

[B52] Pérez-Hernández D. F. (2011). Inventario de los frutales tropicales cultivados tradicionalmente en el T. M. de Santa Cruz de la Palma. [Dissertation thesis]. University of La Laguna, Tenerife.

[B53] Popenoe W. (1959). Avocados in Spain and elsewhere. Cal. Avocado Soc Yearb. 43, 55–66.

[B54] Rodríguez-Hernández Á. Díaz-Díaz R. Zumbado M. Bernal-Suárez M. M. Acosta-Dacal A. Macías-Montes A.. (2022). Impact of chemical elements released by the volcanic eruption of La Palma (Canary Islands, Spain) on banana agriculture and European consumers. Chemosphere 293, 133508. doi: 10.1016/j.chemosphere.2021.133508 34990724

[B55] Ruiz-Chután J. A. Kalousová M. Maňourová A. Degu H. D. Berdúo-Sandoval J. E. Villanueva-González. (2023). Core collection formation in Guatemalan wild avocado germplasm with phenotypic and SSR data. Agronomy 13, 2385. doi: 10.3390/agronomy13092385

[B56] Sandoval-Castro E. Peraza-Magallanes A. Y. Dodd R. S. Ashworth V. E. Cruz-Mendívil A. Calderón-Vázquez C. L. (2021). Exploring genetic diversity of lowland avocado (*Persea americana* Mill.) as a genetic reservoir for breeding. Genet. Resour. Crop Evol. 68, 1–10. doi: 10.1007/s10722-021-01238-w

[B57] Schaffer B. A. Wolstenholme B. N. Whiley A. W. (2013). The Avocado: Botany, Production and Uses (UK: CAB International).

[B58] Schnell R. J. Brown J. S. Olano C. T. Power E. J. Krol C. A. Kuhn D. N.. (2003). Evaluation of avocado germplasm using microsatellite markers. J. Am. Soc Hortic. Sci. 128, 881–889. doi: 10.21273/JASHS.128.6.0881

[B59] Sharon D. Cregan P. B. Mhameed S. Kusharska M. Hillel J. Lahav E.. (1997). An integrated genetic linkage map of avocado. Theor. Appl. Genet. 95, 911–921. doi: 10.1007/s001220050642

[B60] Taddeucci J. Scarlato P. Andronico D. Ricci T. Civico R. Del Bello E.. (2023). The explosive activity of the 2021 tajogaite eruption (La Palma, Canary Islands, Spain). Geochem. Geophys. Geosyst. 24, e2023GC010946. doi: 10.1029/2023GC010946

[B61] Talavera A. Soorni A. Bombarely A. Matas J. A. Hormaza J. I. (2019). Genome-Wide SNP Discovery and genomic characterization in avocado (*Persea americana* Mill.). Sci. Rep. 9, 20137. doi: 10.1038/s41598-019-56526-4 31882769 PMC6934854

[B62] Yangaza I. S. Nyomora A. Joseph C. O. Sangu E. M. Alcaraz M. L. Hormaza J. I. (2024). Genetic diversity and population structure of local avocado (*Persea americana* mill.) from northern Tanzania assessed using SSR markers. Genet. Resour. Crop Evol., 1–19. doi: 10.1007/s10722-024-02246-2

